# SHIP1 is required for T cell surveillance of occult malignancies

**DOI:** 10.1007/s00018-026-06326-7

**Published:** 2026-07-07

**Authors:** S. Fernandes, N. Srivastava, C. Pedicone, R. Sudan, R. W. Engelman, W. G. Kerr

**Affiliations:** 1 Department of Microbiology & Immunology, SUNY Upstate Medical University, Syracuse, NY USA; 2 Department of Experimental Neuroscience, San Martino Hospital IRCCS AOM, Genoa, Italy; 3 Departments of Pathology & Cell Biology and Pediatrics, H. Lee Moffitt Comprehensive Cancer Center and Research Institute, University of South Florida, Tampa, FL USA; 4 Department of Chemistry, Syracuse University, Syracuse, NY USA; 5 Departments of Pediatrics and Microbiology & Immunology, SUNY Upstate Medical University, 750 E. Adams Street, 2204 Weiskotten Hall, Syracuse, NY 13210 USA

**Keywords:** SHIP1/Inpp5d, Tumor immunosurveillance, Intestinal lymphoid tissue, T cells, Histiosarcoma, Epithelial adenocarcinoma, Peyer's patch

## Abstract

SHIP1-deficient mice develop severe, transmural Crohn’s-like ileitis and die prematurely. Recent analysis of a human Crohn’s disease (CD) cohort revealed a significant subset of patients have severely reduced levels of SHIP1 expression suggesting SHIP1 deficiency leads to CD in both rodents and humans. The mucosal inflammatory disease that causes the premature death of SHIP1^−/−^ mice is a consequence of the requirement of SHIP1 expression for the survival of effector T cells in mucosal tissues. T cell specific ablation of SHIP1 expression in CD4CreSHIP1^flox/flox^ mice also results in Crohn’s disease-like ileitis, but less severe than in their germline SHIP1^−/−^ counterparts due to the absence of a SHIP1 deficient neutrophil compartment. Consequently CD4CreSHIP1^flox/flox^ mice live much longer than their SHIP1^−/−^ counterparts. As Crohn’s disease is associated with an increased risk of intestinal cancers in humans and that T cells are proposed to protect from occult malignancies, we hypothesized that CD4CreSHIP1^flox/flox^ mice would develop malignancies originating in the gut. Here we show that CD4CreSHIP1^flox/flox^ mice with ileitis develop spontaneous metastatic cancer originating in the small intestine, including widely disseminated histiocytic sarcoma and intestinal adenocarcinomas. Our findings demonstrate that T cells in the gut are essential to protect against occult intestinal cancers and that SHIP1 is essential for this function. These findings may aid our understanding of the increased susceptibility to intestinal cancer in IBD patients, and particularly since a significant subset of adult IBD patients have defective expression of SHIP1.

**Supplementary Information:**

The online version contains supplementary material available at https://doi.org/10.1007/s00018-026-06326-7.

## Introduction

The SH2-containing 5’ inositol phosphatase 1 (SHIP1) plays divergent roles in cell survival that involve either limiting or promoting activation of the Serine/Threonine kinase Akt/PKB [1, 2]. In addition, SHIP1 is recruited to CD95/Fas in T cells to set a threshold for activation of the Caspase8-mediated extrinsic cell death pathway downstream of CD95/Fas, and particularly for T cells in mucosal tissues [3]. SHIP1 is expressed predominantly in hematopoietic cells, but also in osteoblasts and mesenchymal stem cells [4–6]. Germline deficiency of SHIP1 or induced SHIP1 deficiency in adult mice (where SHIP1 expression is absent in all tissues since the point of conception or after systemic induction of a Cre transgene) results in myeloid infiltration of the lungs and a severe transmural inflammation that is confined to the terminal ileum which closely resembles human Crohn’s disease (CD) [7–10]. Consequently, pulmonary consolidation leads to premature death of germline SHIP1^−/−^ mice within 10–12 weeks of age while severe transmural CD-like ileitis also likely impacts their survival.

Despite extensive evidence for tumor immunosurveillance, its role in controlling spontaneous malignancies arising in chronically inflamed mucosal tissues remains poorly defined. The concept of immune-mediated tumor control was first proposed by Burnet, based on the ability of immune cells to recognize tumor-associated antigens [11]. Thus was born the concept of tumor neo-antigens as a means of tumor recognition and elimination by the immune system. Consequently, T cell surveillance of occult malignancies, and particularly for neo-antigens expressed by the malignant cells, was proposed to protect from cancer, but evidence for this surveillance was confined entirely to carcinogen-treated or oncogene transgenic mice [12, 13]. In addition, tumors were found to occur in genetically immunodeficient mice that could be rejected upon adoptive transfer to wild type (WT) hosts, but not by the homologous mutant strain in which they arose [14, 15]. The immunodeficient strains (RAG2^−/−^, RAG2^−/−^STAT1^−/−^, *PRF1*^−/−^, *PRF1*^−/−^b2m^−/−^) that gave rise to these spontaneous tumors had lost all T or NK cell function, or both, suggesting immune surveillance of spontaneous malignancies requires multiple arms of the immune system [16]. Further evidence that cytotoxic lymphocytes, CD8^+^ T cells and NK cells, may mediate tumor immune surveillance comes from studies of TRAIL- and FasL-mutant mice, which develop spontaneous lymphomas with aging [17, 18]. Consistent with the importance of immune control in FasL-mutant mice, human lymphomas including those that arise in the gastrointestinal tract develop spontaneous mutations in the Fas receptor that presumably enable their escape from immune elimination by FasL-expressing T cells [19, 20]. The immune surveillance theory for tumor control was subsequently modified to account for how the tumor can eventually escape immune control, a process referred to as tumor editing, that includes a three-step progression consisting of tumor *elimination*, *equilibrium* and eventually *escape*.[21] Evidence for immune surveillance of tumors in humans has come largely from studies of melanoma patients, where spontaneous regression of lesions has been correlated with clonal expansion of T cells specific for melanoma epitopes [22–24]. A more recent study provided compelling statistical evidence for active immune surveillance for human colorectal cancers by T cells as mutated peptides that should bind autologous HLA class I molecules are less frequent than expected by chance [25]. The landmark 2010 study showing that immune checkpoint inhibitory antibody administration can be curative in metastatic melanoma provided resounding confirmation that T cell immunity plays a crucial role in cancer control, but importantly also immune escape by tumor cells [26]. These findings suggest that T cells and other cytotoxic lymphocytes contribute to tumor control; however, most evidence derives from systems involving strong oncogenic drivers or profound immune deficiency. Consequently, the extent to which T cell, mediated surveillance limits spontaneous malignancies arising under conditions of chronic inflammation remains unclear.

Given the essential role of SHIP1 in T-cell survival within mucosal tissues, we asked whether loss of SHIP1 in T cells compromises immune surveillance of spontaneous malignancies arising in the inflamed intestine. Here we show that mice with a T cell-selective depletion of SHIP1 expression exhibit severe ileitis as they age. In parallel they develop spontaneous metastatic malignancies that arise in the GI tract. We demonstrate that these malignancies arise due to a failure of conventional TcR-α/β positive surveillance of malignancy as these tumors are largely cleared upon adoptive transfer to WT hosts, but not by TcR-α mutant mice that lack conventional T cell function. Our findings demonstrate a critical role for T cells in surveilling occult malignancies in the gut in the absence of oncogenic or mutation drivers of malignancy and identify SHIP1 as essential for this T cell effector function.

## Results

### **T cell-specific SHIP1 deficiency drives age-dependent Crohn’s-like Iieitis in mice**

**Fig. 1:**
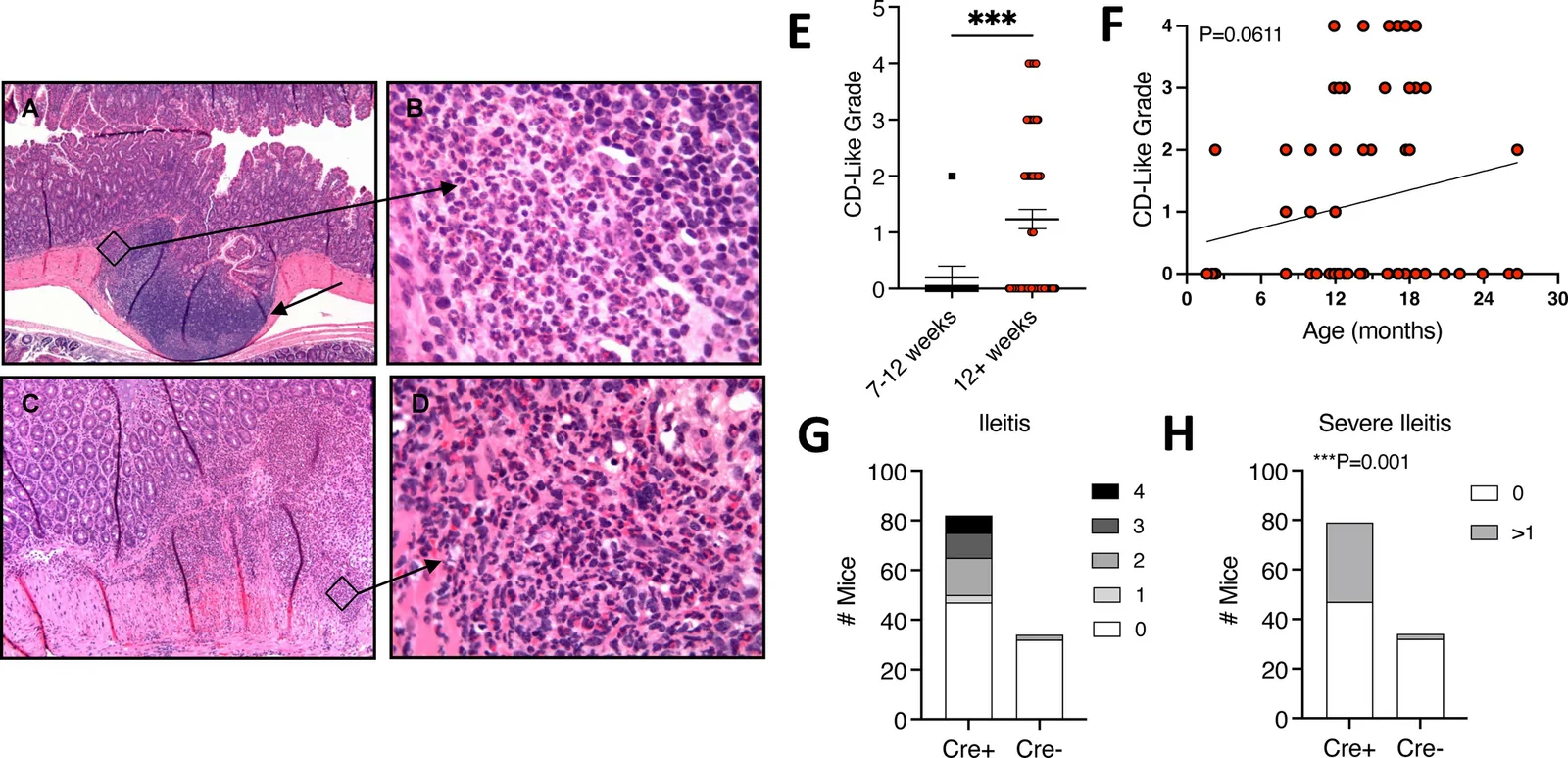
Ileitis occurs in mice that lack expression of SHIP1 in T cells. Histopathological analysis of the ileum of CD4CreSHIP^flox/flox^ mice that develop Crohn’s disease-like ileitis comprised of a marked, mixed leucocyte infiltration, here predominantly polymorphonuclear, and in some cases of the lamina propria and underlying lymph nodule (**A**, **B**) consistent with inflammatory grade 2, and in others extending deep into the tunica muscularis (**C**, **D**) consistent with inflammatory grade 4. (**E**) Comparison of the incidence of ileitis in the ilieum young vs old adult CD4CreSHIP1^flox/flox^ mice (Welch’s T-test, two-tailed)., ***, *p* < 0.001. (**F**) Linear regression analysis of the incidence of ileitis vs age. (*p*-value, likelihood ratio test) (**G**) Incidence of ileitis (by severity score) in CD4CreSHIP1^flox/flox^ and SHIP^flox/flox^ littermates. (**H**) Incidence of severe ileitis (> grade 1) in CD4CreSHIP1^flox/flox^ and SHIP^flox/flox^ littermates (*p*-value, Fisher’s Exact test). No inflammatory disease was noted in other regions of the small intestine or in the large intestine of CD4CreSHIP1^flox/flox^ mice

We previously reported that germline SHIP1^−/−^ mice exhibit a profound depletion of both CD4^+^ and CD8^+^ T cells in the small intestine (SI) with a concomitant increase in neutrophils that penetrate the lamina propria of the small intestine [7]. We noted a similar decrease in both CD4^+^ and CD8^+^ T cells in the SI and lungs of young adult CD4CreSHIP1^*flox/flox*^ mice (2–3 months); however, we did not observe a significant incidence of ileitis in these mice [3]. We then performed gross and histopathological examination of the small and large intestines of aged CD4CreSHIP^*flox/flox*^ mice (≥ 8 months of age) and this revealed numerous enlarged reactive lymph nodules with prominent germinal centers with 71% (17 of 24) of these aged mice exhibiting a CD-like ileitis comprised of moderate, mixed, predominantly polymorphonuclear leukocyte infiltration. We show examples of moderate grade 2 (Fig. 1A, B) and severe grade 4 ileitis (Fig. 1C, D) in these aged CD4CreSHIP^*flox/flox*^ mice. This age-dependent pathology onset is underscored by minimal ileitis in young adults (1/12 mice at 7–12 weeks; Fig. 1E), confirming significantly elevated risk with aging. Linear regression analysis revealed no further severity increase beyond 8 months (Fig. 1F). Cumulatively, we find that CD4CreSHIP1^flox/flox^ mice exhibit both a significantly increased incidence of ileitis (*p* < 0.001) (Fig. 1G) and increased incidence of moderate to severe ileitis (grade 2 or higher, *p* = 0.0005) (Fig. 1H) as compared to SHIP1-sufficient, SHIP1^flox/flox^ littermates. Thus, T cell-specific SHIP1 deficiency induces age-dependent Crohn's-like ileitis in mice, with significantly higher incidence and severity in aged mice (≥ 8 months) as compared to young adults or SHIP1-sufficient controls.

### ***A SHIP1-deficient T cell compartment fails to detect and eliminate spontaneously arising cancers in the gastrointestinal tract***

**Fig. 2:**
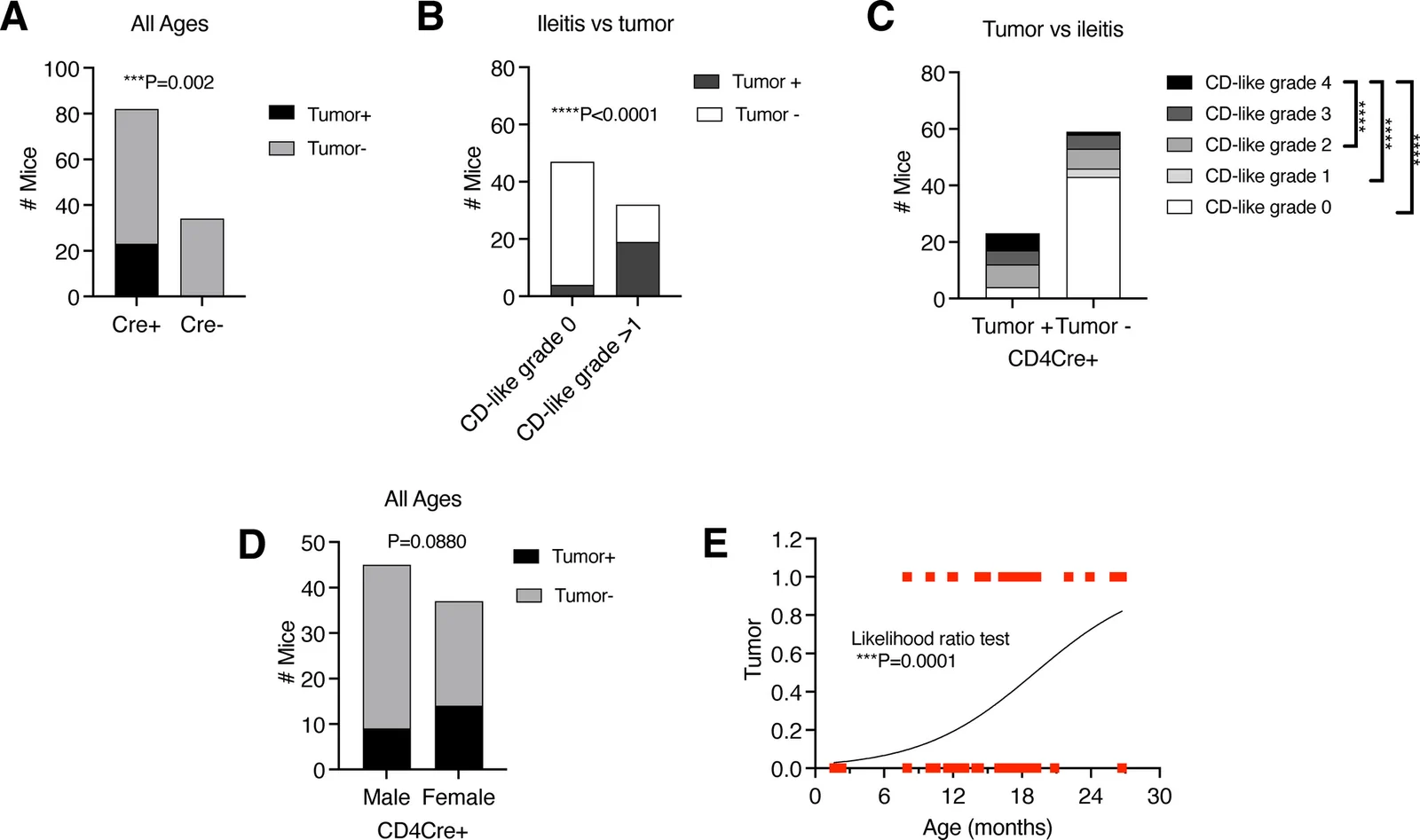
Mice with T cell selective SHIP1-deficiency develop spontaneous intestinal malignancies. (**A**) Frequency of malignancy detection in CD4CreSHIP1^flox/flox^ mice vs. age-matched SHIP1^flox/flox^ controls.(*p*-value, two-sided Fisher’s exact test) (**B**) Frequency of malignancy formation in CD4CreSHIP1^flox/flox^ mice with moderate-to-severe ileitis (grade > 1) vs without (grade 0).(*p*-value, two-sided Fisher’s exact test) (**C**) Frequency of malignancy formation in CD4CreSHIP1^flox/flox^ mice with different grades of ileitis (grades 1–4) vs. without (grade 0).(*p*-values, two-sided Fisher’s exact test) (**D**) Frequency of malignancy detection in male and female CD4CreSHIP1^flox/flox^ mice.(*p*-value, two-sided Fisher’s exact test), (**E**) Likelihood of malignancy formation increases with age in CD4CreSHIP1^flox/flox^ mice. (*p*-value, Simple logistic regression with likelihood ratio test)

**Fig. 3:**
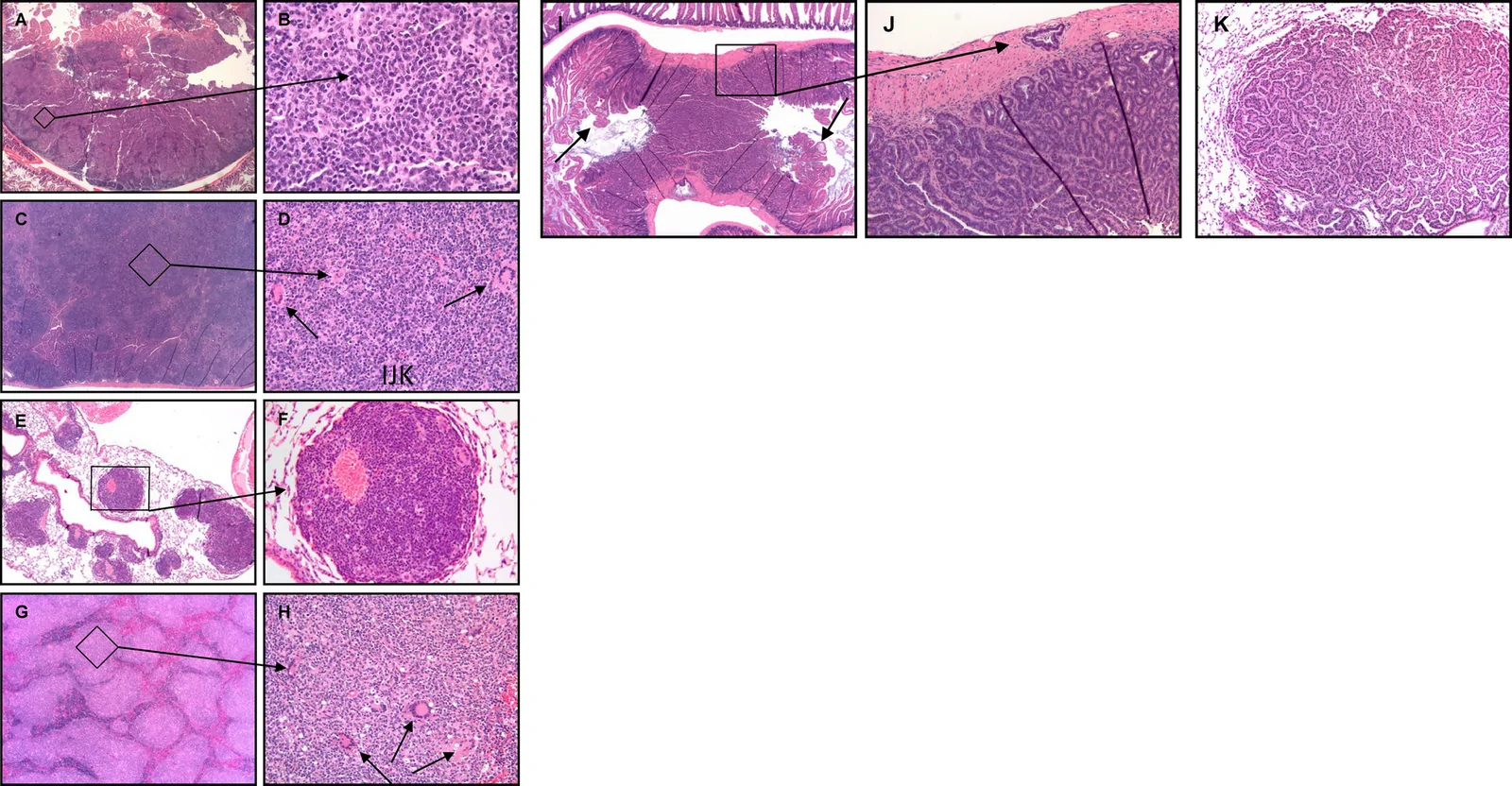
Mice with T cell selective SHIP1-deficiency develop spontaneous histiocytic sarcomas or small intestinal adenocarcinomas. (**A**-**H**) CD4CreSHIP1^flox/flox^ mice develop widely disseminated histiocytic sarcoma, originating within the CD like ileitis, which is locally invasive and distorts intestinal architecture (**A**) and is comprised of large histiocytic cells with pale eosinophillic cytoplasm (**B**), or effaces intestinal architecture (**C**) and is comprised of pleomorphic histiocytes with frequent multinucleated giant cells (**D**, arrows), and disseminates to mesenteric lymph nodes, lung (**E**, **F**), and spleen (**G**) with the splenic red pulp filled with neoplastic histiocytes and frequent multinucleated giant cells (**H**, arrows). Some CD4CreSHIP1^flox/flox^ mice develop small intestinal adenocarcinomas comprised of pleomorphic papillary fronds and neoplastic glands of hyperchromatic, dysplastic epithelium that extend into the lumen (**I**, arrows), penetrate the tunica muscularis (**J**), and metastasize to the lung (**K**)

Chronic inflammation in the small intestine is associated with increased risk of cancer in patients with inflammatory bowel disease [27–30]. We hypothesize that T cell deficiency driven-ileitis in CD4CreSHIP1^flox/flox^ mice may similarly predispose these mice to gastrointestinal malignancies. To examine this possibility, we subjected aged CD4CreSHIP^flox/flox^ (≥ 8 months of age) mice to systematic necropsy to assess the presence of occult malignancy. The small intestine, large intestine, mesenteric lymph node, spleen and lungs were examined grossly and microscopically. Of 82 CD4CreSHIP^flox/flox^ mice that were examined 23 (28%) had developed malignant tumors, while no malignancies were present in age-matched SHIP^flox/flox^ littermate controls (Fig. 2A). Thus mice with a selective SHIP1 deficiency in the T cell compartment have a significantly increased incidence of not only developing ileitis, but also malignancy in the small intestine. Indeed, malignancy was much more likely to be present in CD4CreSHIP^*flox/flox*^ mice that also had ileitis (*p* < 0.0001) (Fig. 2B). In addition, there was also significantly increased incidence of tumor formation in mice that had moderate to severe ileitis (grades 2–4) (Fig. 2C), supporting an association between IBD and malignancy development in these mice. Importantly, each tumor in all the affected CD4CreSHIP1^flox/flox^ mice appeared to originate from within the inflamed small intestine suggesting that loss of T cells surveillance at a mucosal site undergoing constant exposure to commensal microbiota and associated inflammatory stimuli might contribute to the development of malignancy. The frequency of spontaneous tumor formation was not significantly different in male and female CD4CreSHIP1^flox/flox^ mice (Fig. 2D). Unlike with ileitis, we did, however, find a significant correlation between aging and the incidence of tumor development in CD4CreSHIP1^flox/flox^ mice (*p* = 0.0038) (Fig. 2E). Tumors were primarily histiocytic sarcomas that were widely disseminated to the mesenteric lymph nodes, spleen and lung (Fig. 3A-H). In addition, two small intestinal adenocarcinomas that were locally invasive to the tunica muscularis and that metastasized to the lung (Fig. 3I-K) were also identified in CD4CreSHIP1^flox/flox^ hosts. In addition to the primary malignancies present in the intestines (Fig. S1A), multiple metastases were present in CD4CreSHIP1^flox/flox^ mice, including in the draining mesenteric lymph node, spleen, lung, thymus, liver and salivary glands (Fig. S1B,C). Collectively, our findings demonstrate that T cell-intrinsic SHIP1 deficiency leads to age-dependent spontaneous tumorigenesis and metastasis driven by chronic ileitis.

### ***Histiosarcomas consisting of macrophage-like cells are aboundant in the Peyer’s Patch and at sites of metastasis***

**Fig. 4:**
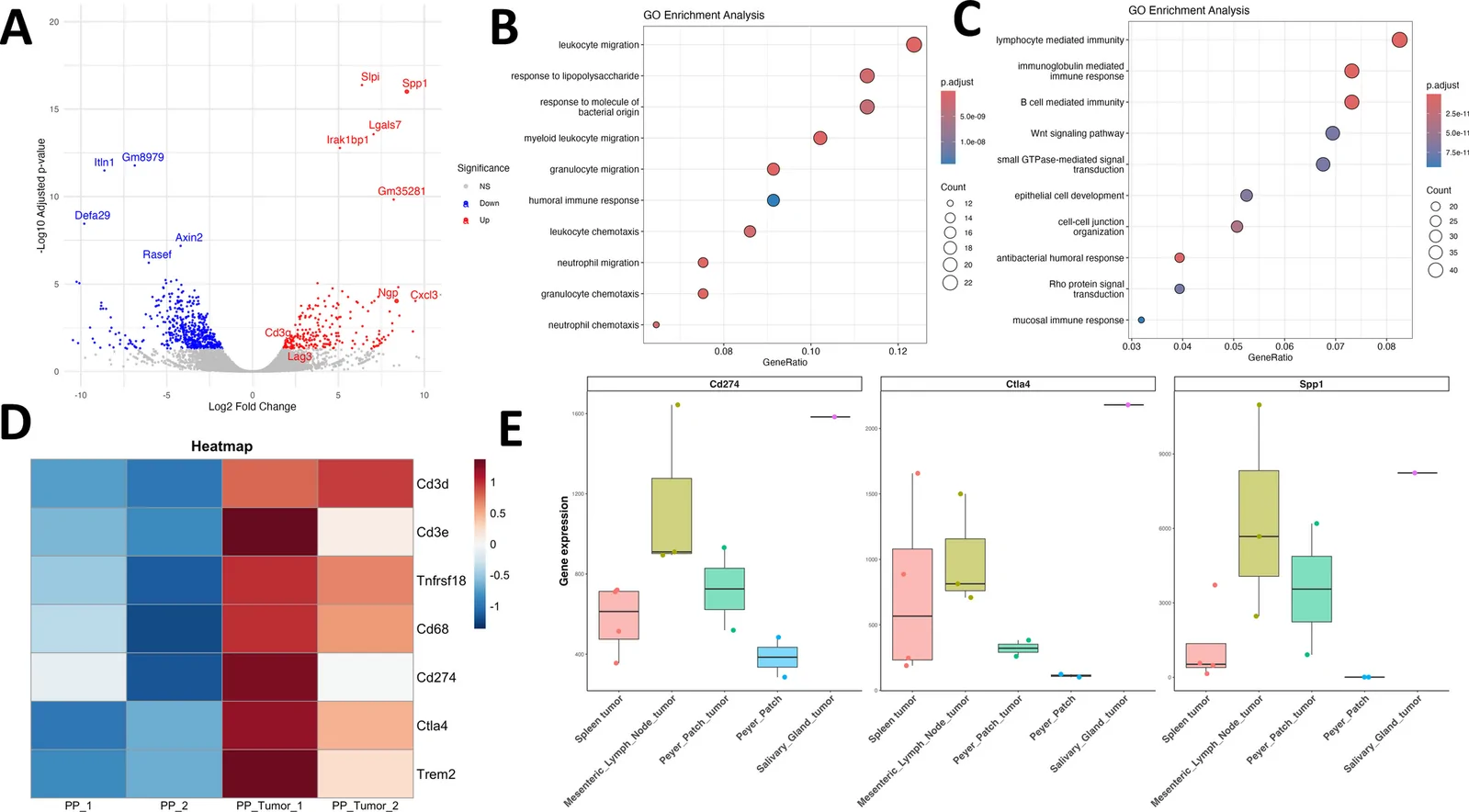
Transcriptome analysis indicates histiocytic sarcomas and their metastases are composed largely of an immunosuppressive macrophage population along with increased Treg cells. (**A**) Volcano plot showing top dysregulated genes (padj < 10E^−6^) with significant genes highlighted (Log2FC > 1 in red, Log2FC < −1 in blue) for the comparison PP tumor vs control tissue with 2 mice per condition. (**B**) Upregulated and (**C**) downregulated GO Molecular Function pathways derived from the respective significant DEG in PP. (**D**) Heatmap of selected genes related to immune function and macrophage genes in comparison PP primary tumors versus normal PP tissue. (**E**) Immunomodulatory genes upregulated at sites of metastasis (4 Spleens, 3 Mesenteric Lymph nodes, 1 Salivary Glands) as compared to normal PP and PP histiocytic tumors

We performed bulk RNAseq analysis of multiple tumors harvested from CD4CreSHIP1^flox/flox^ immune organs including the Peyer’s patch (PP). We compared the transcriptome of tumor-containing PP in CD4CreSHIP1^flox/flox^ mice to normal PP in order provide a molecular signature for tumor cells present in the originating tissue, but also genes that might be preferentially up-regulated in these PP tumors. Principal component variance analysis showed most of the variance is explained by the first 5 PCs (Figure S2A,B), all minimally affected by sex, mouse or tissue that contained the tumor, while PC2 was significantly correlated with tumor presence (Status) (Figure S2C). This analysis revealed that tumor tissue was populated by a well-differentiated myeloid phenotype that express markers representative of terminally-differentiated macrophages. For instance CD68, SPP1/osteopontin, Ngp/Neutrophilic granule protein, CXCL3, Dectin1/Clec7A and TREM2 expression were present and in some cases were increased in PP tumors (Fig. 4A, D). Pathway analysis revealed upregulated leukocyte migration and response to LPS/humoral response genes (Fig. 4B), while the top downregulated pathway featured gene expression associated with lymphocyte mediated immunity (Fig. 4C). Interestingly, both SPP1 and CXCL3 were increased in the transcriptome of tumor-bearing Peyer’s Patches (Fig. 4A). CXCL3 is an inflammatory chemokine secreted by tumor-resident macrophages that promotes immune escape and metastasis of tumor cells [31]. Other genes found to be upregulated in PP tumors were Lag3, Ngp and the CD3g receptor subunit (Fig. 4A). Other T cell receptor subunits (e.g. CD3e, CD3d), although upregulated, did not reach significance when performing a multiple correction analysis of the data (Fig. 4D). Like CXCL3, SPP1 (osteopontin) expression is also up-regulated in PP tumors. SPP1 is highly-expressed in tumor macrophages and promotes tumor growth and invasion [32]. Recently the CXCL9:SPP1 ratio has been found to be a strong indicator of macrophage polarization in human tumors and with a particularly poor prognosis that is correlated with the frequency of SPP1^hi^ tumor-associated macrophages (TAM) while the presence CXCL9^hi^ TAM was associated with improved survival [33]. SPP1 also has potent immunoregulatory activity on T cells in tumors [33]. Consistent with this we note high expression of CD274 (PDL1) and CTLA4 in PP tumors and particularly at sites of metastasis like mesenteric LN, spleen and salivary gland and this is correlated with increased SPP1 in these PP tumors and sites of metastasis like mesenteric LN and spleen (Fig. 4D, E). We also observe increased expression of Lag3 in PP tumors suggesting there is a T cell suppressive microenvironment in PP organs where tumors arose (Fig. 4A). Thus, these tissue macrophage-like tumors and their metastases might also harbor a prominent Treg population that likely suppresses effector T cell responses to tumor neoantigens. Recently, CD4^+^ Tr1 cells have been shown to eliminate neo-antigen expressing tumor cells [34] and this T cell subset exhibit increased expression of CCL5 and granzymeB, two genes that we also found to be increased in PP tumors (Fig. 4A). Taken together transcriptome analysis of tumors in CD4CreSHIP1^flox/flox^ hosts identify the presence of macrophage-like cells with increased expression of genes that are immunosuppressive and that can facilitate metastasis (SPP1, Lag3, Ngp, CXCL3). RNAseq analysis of the PP histiocytic tumors also showed that there was no diminished expression of SHIP1/Inpp5d mRNA in PP tumors vs. normal PP tissue indicating there has been no spurious deletion at the Inpp5D locus leading to loss of SHIP1/Inpp5d expression in these histiosarcomas (Fig. S2D).

To further define the tumor population we performed spectral flow cytometry on spleens from histiocytic sarcoma bearing animals (Fig. S3A) and this revealed a larger frequency of CD3^−^CD19^−^ (non-B/T) cells (Fig. S3B) that exhibit increased representation of CD11b^+^F4/80^+^Gr1^−^ cells indicative of tissue-resident macrophages that can be found in intestinal tissues like PP [35]. Intracellular flow cytometric analysis of SHIP1 protein expression in one of the sarcomas confirmed that SHIP1 protein expression is ablated in T cells present in a histiocytic sarcoma, but not in CD11b^+^F4/80^+^Gr1^−^macrophage subset that is increased in the histiocytic sarcomas.(Fig. S3C) Taken together analyses of both SHIP1/Inpp5d mRNA and protein expression support that histiocytic sarcomas are unlikely to arise from spurious expression of the CD4Cre transgene and SHIP1 deletion in myeloid lineage cells.

### ***Spontaneous malignancies that arise in mice with a SHIP1-deficient T cell compartment can be eliminated in hosts with a normal T cell compartment***

**Fig. 5:**
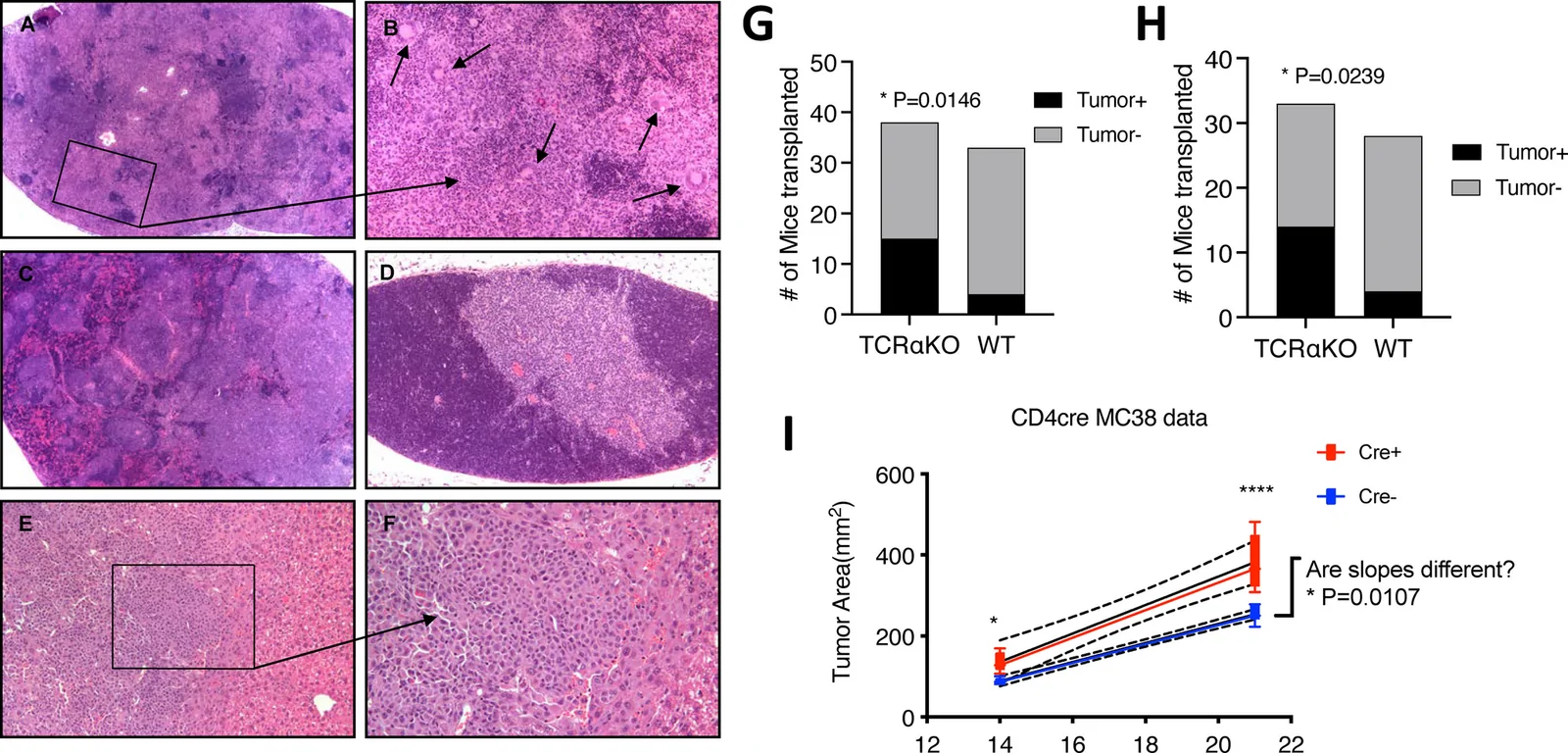
SHIP1-sufficient T cell compartment can mediate control of spontaneous malignancies that arose in CD4CreSHIP1^flox/flox^ hosts. Tumor-bearing tissues were harvested and a single cell suspension from these tissues was then adoptively transferred to TcR-a^−/−^ or WT (C57BL/6) hosts. Tissues from the transplanted recipients (spleen, mLN, liver, SG, SI, large intestine, lung) were then harvested 10–14 weeks post-transplant and scored by H&E for tumor growth. TcR-a^−/−^ mice that showed tumor growth typically developed widely disseminated histiocytic sarcoma, which is invasive and distorts or effaces the architecture of the lymph node (**A**), spleen (**C**), thymus (**D**), and/or liver (**E**), and is comprised of pleomorphic histiocytes (**B**, **F**), often with frequent multinucleated giant cells (**B**, arrows). Statistical assessment of tumor growth in TcR-a^−/−^ vs. WT recipients: (**G**) 2.5 × 10^6^ cells (derived from 8 different tumors from 6 different CD4CreSHIP1^flox/flox^ hosts) or (**H**) 5 × 10^6^ cells (derived from 7 different tumors from 5 different CD4CreSHIP1^flox/flox^ hosts) were injected (i.v.) into 3–7 TcR-a^−/−^ or 4–7 WT mice for each tumor. *P*-values, two-sided Fisher’s Exact Test. (**L**) MC38 tumor volume at days 14 and 21 following subcutaneous injection of 1X10^6^ tumor cells in hosts of the indicated genotype (*, *p* < 0.05, **** *p* < 0.001, two-way ANOVA with Holm-Sidak correction for group analysis of repeated measures. Linear regression for slope analyses *, *p* < 0.05)

We then investigated whether intestinal cancers arising in CD4CreSHIP1^flox/flox^ mice could be eliminated by hosts possessing a functional, SHIP1-sufficient T cell compartment. Previous studies have shown that growth of lymphomas which spontaneously arose in mice could be effectively controlled by mice with a normal T cell compartment but not by mice with a perforin mutation that impacts the function of both cytolytic T cells and NK cells [15]. However, we chose to test this in mice that have a more targeted immune defect, specifically in mice that lack functional TcR-α/β^+^ T cells. To test this we adoptively transferred an equal number of cells from multiple independent histiocytic sarcoma-bearing tissues derived from different CD4CreSHIP1^flox/flox^ mice into either TcR-α^−/−^ or WT hosts (Fig. 5). We found that the majority of the WT hosts (with a SHIP-sufficient T cell compartment) eliminated tumors, whereas a high proportion of TcRα^−/−^ recipients developed widely disseminated histiocytic sarcoma growth in multiple organs (Fig. 5A-F). The frequency of TcRα^−/−^ mice showing tumor engraftment and growth was significantly higher when either 2.5 × 10^6^ (Fig. 5G) or 5 × 10^6^ cells (Fig. 5H) from histiocytic sarcomas from CD4CreSHIP1^flox/flox^ mice were adoptively transferred to TcRα^−/−^ hosts. Thus, the histiocytic sarcoma cells which arise in CD4CreSHIP1^flox/flox^ hosts can be eradicated by hosts that have a SHIP1-sufficient TcR-α/β^+^ T cell compartment. To provide further evidence of defective cancer immunosurveillance by SHIP1-deficient T cells, we also compared tumor growth of syngeneic colon adenocarcinoma (MC-38) cells in CD4CreSHIP1^flox/flox^ vs. SHIP1^flox/flox^ mice. MC-38 cells express numerous tumor neo-antigens that make them amenable to T cell control[36] We found that CD4CreSHIP1^flox/flox^ mice are significantly more permissive for growth of MC38, with a significant increase in tumor volume at 14 and 21 days, but also an increased speed of tumor growth (slope) (Fig. 5I). Taken together, these studies demonstrate a novel role for SHIP1 in immune function - specifically the ability of SHIP1-expressing conventional T cells to control and/or eliminate malignant cells and particularly for occult malignancies that arise spontaneously in the gastrointestinal tract.

## Discussion

Here we demonstrate that T cell specific SHIP1 deletion increases susceptibility to spontaneous tumorigenesis, with preferential development in intestinal lymphoid tissues. Importantly, this study identifies a previously unrecognized role for SHIP1 in T cell–mediated immune surveillance of spontaneous malignancies, and supports CD4CreSHIP1^flox/flox^ mice as a model of age-dependent, inflammation-associated tumorigenesis arising in the absence of engineered oncogenic drivers. The malignancies that arise in CD4CreSHIP1^*flox/flox*^ mice are largely eradicated upon transfer to WT, but not TcRα^−/−^ hosts, indicating the increased susceptibility to malignancy in CD4CreSHIP1^*flox/flox*^ mice is consistent with a reduction in effector T cell-mediated immune surveillance. The loss of T cell surveillance in CD4CreSHIP1^*flox/flox*^ mice could be due to the fact that SHIP1-deficient T cells in the gut are more susceptible to apoptosis as SHIP1 is recruited to CD95/Fas to limit Caspase8-mediated T cell apoptosis at mucosal sites [3]. Additionally in CD4CreSHIP^*flox/flox*^ mice regulatory T cells are increased in all lymphoid and non-lymphoid tissues [3], which during aging may further suppress effector T cell function to promote tumor initiation. Indeed, RNAseq analysis of tumor-bearing organs in CD4CreSHIP1^flox/flox^ indicates increased expression of an immunosuppressive gene cassette (LAG3, CTLA4, PD-L1). Importantly, tumor incidence was only seen in the T cell specific knock out of SHIP1 and no incidence of tumor formation has been observed in aged myeloid specific knockout of SHIP1, LysMCreSHIP^flox/flox^ mice,(data not shown), further suggesting that the histiocytic sarcomas develop as a result of compromised T cell immune surveillance for occult malignancies. Interestingly, we did observe that a minor subset of histiocytic sarcomas that arose in CD4CreSHIP^*flox/flox*^ mice were able to avoid immune control in WT hosts. This could be due to immune escape as these macrophage-like tumors appear to express an array of potently immunosuppressive molecules, including LAG3, CTLA4 and PDL1 (CD274).

We previously found that effector T cells, and particularly CD4 + T cells, are diminished in the small intestine of SHIP1 KO mice which we proposed was exacerbated by neutrophilic infiltration of the ileum that correlated with severe ileitis [7]. Subsequent analysis in CD4CreSHIP1^flox/flox^ mice showed that the reduction of CD4^+^ T cells in mucosal tissues was due to Fas-mediated cell death which SHIP1 limits [3]. Conversely, SHIP1 limits CD4 + Treg cell homeostasis and function [37, 38], including in the small intestine [3]. Our findings here in older CD4 + CreSHIP1^flox/flox^ mice suggest there could be a price to pay for the selective survival of SHIP1-deficient Treg cells in gut lymphoid tissue as the transcriptome of SHIP1-deficient PP tumors and widely disseminated lesions consistent with metastatic spread shows increased representation of T cells with increased LAG3, CTLA4 and PD-L1. These Treg cells would be expected to be quite potent suppressors of effector T cells responses that might eradicate nascent malignancies such as the histiocytic sarcomas that arise in the PP of CD4CreSHIP1^flox/flox^ mice. Alternatively, or possibly in addition to, SHIP1 may also limit the recently described CD4^+^ Tr1 cell population that kills CD4^+^ effector T cells that participate in control of occult malignancies [34], as we also observe increased expression of genes selectively expressed by this CD4^+^ Tr1 cell subset in histiocytic sarcomas and their metastases. CD4CreSHIP1^flox/flox^ mice represent a model of age-dependent, spontaneous, inflammation-associated tumorigenesis, in which malignancies arise in the absence of genetic or chemical drivers of epithelial transformation. Future studies with in depth immunophenotyping and single-cell analysis of the tumors, neoantigen studies and phylogenetic analysis of the metastases will be necessary to fully define mechanisms that underly this impairment of T cell function that leads to immune escape of occult malignancies, currently limited in this study. A limitation of this study is that the relatively small datasets used for flow cytometry and RNA-seq provide only an initial characterization of the immune landscape. Future studies with larger cohorts and more comprehensive immune profiling of both metastases and primary tumors may help to further define the expanded CD11b^+^F4/80⁺Gr1⁻ populations present in histiosarcomas and other innate immune subsets potentially associated with NGP upregulation we observed in the Peyer Patch tumors.

We have earlier shown that the selective demise of T cells in the small intestine contributes to inflammatory bowel disease (IBD) in SHIP1 deficient mice [3]. It has been shown that chronic IBD is associated with increased risk of intestinal cancer [28–30]. In previous studies of human IBD patient populations, we also found that a significant subset (~ 15%) exhibit a profound degree of SHIP1 deficiency at the level of protein expression (10% of normal) [39, 40]. We found that SHIP1 deficiency resulted from dysregulation of ubiquinases or deubiquinases that fine tune the steady state expression of SHIP1 protein via the proteasome [40, 41]. Interestingly, like CD4CreSHIP1^*flox/flox*^ mice the subset of IBD patients that exhibit an ~ 90% reduction in SHIP1 expression also show reduced number of circulating CD4^+^ T cells [40]. Moreover, the SHIP1-deficient subset of IBD patients had more severe inflammatory disease requiring multiple surgical resections of the intestine, but also a higher incidences of intestinal cancer as compare to SHIP1-sufficient IBD patients [40]. A similar phenotype has been described in SHIP1^−/−^ mice and mice with inducible SHIP1 deletion in all lineages [7]. However, these mice develop more severe colitis and do not survive more than 2 months due to eosinophilic consolidation of the lungs which prevents the assessment tumor development with age. Taken together, our current findings and our previous analysis of CD4^+^ T cell numbers, cancer incidence and SHIP1 expression in IBD patients suggest the role of SHIP1 in T cell homeostasis and control of occult malignancies in the gut might be conserved in both species.

Our findings offer an insight leading to a possible therapy for the subset of IBD patients who are SHIP1-deficient and suffer from very severe disease including intestinal malignancy. We and others have developed small molecule agonists of SHIP1 that enhance its catalytic activity (AQX-MN100, K306) [42, 43]. However, the SHIP1-deficient subset of IBD patients retain some degree of SHIP1 protein expression, albeit at greatly reduced levels relative to healthy controls [39, 40]. It is possible that even partial restoration of SHIP1 activity in T cells resident in the gut could partially rescue T cell function and this could be sufficient to ameliorate severe inflammatory disease in the intestine, and thus, based on our findings here, prevent the development of malignancies. As inflammatory disease in both the lung or gut has not been observed in SHIP1 haplo-insufficient mice (SHIP^±^) [7], it seems plausible that only a partial rescue of SHIP1 protein activity by an agonist might be an effective therapeutic strategy in SHIP1-deficient IBD patients.

## Materials and methods

### **Mice**

CD4CreSHIP^flox/flox^ mice are on C57BL/6 background and were created by intercrossing CD4Cre purchased from Jackson laboratory and SHIP^flox/flox^ mice as described previously. Mice were housed in specific pathogen free conditions excluding Murine Norovirus (MNV), Helicobacter sp., Syphacia spp., Aspiculuris tetraptera, Parainfluenza virus Type 1 (Sendai), Coronavirus (MHV), Mycoplasma pulmonis, Paramyxovirus (PVM), Parvovirus (MVM and MPV), Poliovirus (TMEV-GDVII), Reovirus Type 3, Lymphocytic Choriomeningitis Virus, Mouse Adenovirus types 1 & 2, Poxvirus (Ectromelia), Rotavirus (EDIM), Papovavirus (Poly), Hantaan Virus, Cilia-associated Respiratory Bacillus, Clostridium piliforme (Tyzzer’s), Encephalitozoon cuniculi and Corynebacterium bovis.

### **Histopathological analysis and CD score**

We have previously described procedures for the histopathological analysis of inflammation and malignancy in mouse intestinal and immune tissues.[3, 7, 9]. Briefly, the gut and lungs were insufflated with 10% neutral-buffered formalin, and the small intestines (SIs) were arranged as segmented Swiss rolls to fit into cassettes for histologic processing. Tissues were fixed in 10% neutral-buffered formalin, dehydrated, paraffin embedded, sectioned at 3 µm, and stained with hematoxylin and eosin. Inflammatory lesions were graded as follows: grade 0, no significant abnormalities; grade 1, mild predominantly polymorphonuclear (PMN) leukocyte infiltration (< 25 PMNs/high-power field [hpf]) within the lamina propria and/or enteric lymphoid nodules; grade 2, moderate predominantly PMN leukocyte infiltration (> 25 cells/hpf) within the lamina propria and/or lymphoid nodules; grade 3, marked inflammatory cell infiltration extending below the muscularis mucosa with associated distortion of the mucosal and submucosal architecture and crypt hyperplasia; grade 4, marked infiltration extending into the tunica muscularis; grade 5, marked transmural leukocyte infiltration; and grade 6, marked transmural leukocyte infiltration with extension into the mesentery and/or adjacent organs. A veterinary pathologist diagnosed the tumors as histiocytic sarcoma due to their aggressive disseminated behavior and characteristic histologic features, including pleomorphic histiocytic cells with abundant eosinophilic cytoplasm and multinucleated giant cells infiltrating and effacing the normal architecture of the intestine, lymphoid organs, and lungs.

### **RNA seq analysis of tumor transcriptome**

A total of 9 mice were used in this study. Sex, age, and mouse-of-origin information are included in Table S1. Most tumors were collected from different mice; only mice 3, 5, and 6 contributed more than one tumor sample. All Peyer’s patch tumor samples and control Peyer’s patch samples came from different mice. RNA was prepared with Qiagen RNAeasy kit from fresh frozen tumors, metastases and control tissue and submitted to Novogene for library prep with polyA enrichment and sequencing 2 × 150 bp 40 M reads sample. Alignment to GRCm38 was performed with STAR (v2.5), while HTSeq v0.6.1 was used to map reads to genome. From raw counts genes in X and Y chromosome were removed before performing differential expression analysis in R 4.3.2 with DeSeq2 1.42.1 and plotted results with ggplot2 3.5.2. Gene Ontology (GO) enrichment analysis of differentially expressed genes was performed using the Biological Processes terms from org.Mm.eg.db 3.18.0 with clusterprofiler 4.10.1. Significance was assessed with multiple corrections using the BH method. Raw and processed files have been submitted to GEO (Accession number: GSE313296). The R pipeline used to perform the above analysis has also been deposited on GitHub.

## Spectral flow cytometry

Spleens were mechanically dissociated over a 70um filter with 15 mL Cold Flow Buffer (PBS + 3% FBS + 1 mM Hepes). Samples were centrifuged for 5 min at 350xg, decanted and resuspended in 5 mL of 1X RBC lysis buffer (Cat # 00–4333-57, ThermoFisher Scientific). After 5 min incubation with occasional vortexing, samples were diluted to 20 mL with PBS and centrifuged 5 min at 350xg and decanted. Samples were resuspended, counted and adjusted to 10 × 106cells/mL in flow buffer. 5 M cells per sample were washed and resuspended in 100uL PBS. Samples were stained with 0.3uL/tube of 1 × Zombie Aqua Fixable Viability Stain for 15 min at 4 °C. Samples were washed with 2 mL cold PBS and centrifuged 5 min at 350xg and decanted. Fc receptors were then blocked with 2uL of TruStainFcX (Cat # 101320, BioLegend) for 15 min at 4 °C. Samples were then stained with the antibody cocktail (BV421 anti-CD21/35, BV605 anti-Gr1, BV650 anti CD11c, BV785 anti-B220, Alexa fluor 700 anti-Ly6C, Biolegend, V450 anti-CD4, BV711 anti-CD11b, PerCP anti -CD45, PE-CF954 anti-CD3e, PE-Cy7 anti-CD19 APC-Cy7 anti-CD8a, BD biosciences, and FITC anti-MHCII, CD90.2 PE-Cy5, PerCP-efluor710 anti-CD117 AlexaFluor 647 anti-F4/80, ThermoFisher Scientific) for 30 min at 4 °C. Samples were washed with 2 mL cold PBS and centrifuged 5 min at 350xg and decanted. Washing step was repeated and cells fixed with 1 × IC Fixation buffer (ThermoFisher Scientific) for 20 min. Samples were washed twice with 2 mL 1X Permeabilization Buffer (ThermoFisher Scientific) and were resuspended in 100uL 1X Permeabilization Buffer with PE-Anti-SHIP1 (Biolegend) for 30 min at 4 °C followed by 2 mL washing in 1X Permeabilization Buffer and centrifugation at 400xg for 5 min prior to decanting. Samples were resuspended in 1 × Perm Buffer and acquired on the Cytek Aurora. Fluorescence minus one (FMO) control samples, and Dead cell control sample were stained simultaneously using sample along with an unstained cell control. UltraComp Plus Compensation beads (BD) were singly stained with each antibody and used for the acquisition of single stained samples. Data was unmixed on SpectroFlo and exported as Raw and Unmixed FCS files. Data was further analyzed with FlowJo 10.9.0, and statistical analysis was performed with GraphPad Prism.

## Supplementary Information

Below is the link to the electronic supplementary material.

Supplementary file1 (XLSX 7448 KB)

Supplementary file2 Fig.S1. Photographic images of different primary and metastatic tumors isolated from CD4CreSHIP1flox/flox mice. (A) Small intestine, (B) Mesenteric lymph node (mLN) and a normal mLN, (C) A tumor bearing spleen and normal spleen, (D) Lung, (E) Thymus, (F) Liver, (G) Two examples of salivary gland tumors and a normal salivary gland. Normal tissues are shown for contrast for mLN, spleen and salivary gland. Fig.S2. RNA seq QC and analysis of SHIP1 expression in PP tumors and cells. (A) Principal component analysis of RNA eq dataset (B) PC1 and 2 displayed by sex. (C) Correlation analysis of 5 top PC with Sex, Mouse, Tumor status and Tissue. (D) SHIP1 mRNA expression in control tissue (normal Peyer’s Patches) and Peyer’s Patches with histiosarcomas. There was no decrease observed for SHIP1 mRNA expression in the tumors vs. normal PP controls. Fig. S3. Flow cytometry tumor analysis (A) Gating used for populations in Cre- and Cre+ mesenteric lymph nodes used for defining population percentages of Live cells for of B,T and myeloid cells in tumor or normal tissue. (B) Percentage of live for B, T cells, Myeloid and Cd11b+F480+Gr1- gated as in A (C) Intracellular flow cytometry for SHIP1 protein expression in LN from three Cre+ and three WT mice with FMO stain for each population. (PDF 10572 KB)

## Data Availability

All raw data is available upon request. The RNAseq datasets have been deposited on GEO.

## References

[1] Krystal G (2000) Lipid phosphatases in the immune system [In Process Citation]. Semin Immunol 12(4):397–403. [Online]. 10.1006/smim.2000.0222 10995586

[2] Kerr WG (2011) Inhibitor and activator: dual functions for SHIP in immunity and cancer. Ann N Y Acad Sci 1217:1–17. 10.1111/j.1749-6632.2010.05869.x PMC4515353 21155837

[3] Park MY et al (2014) Impaired T-cell survival promotes mucosal inflammatory disease in SHIP1-deficient mice. Mucosal Immunol 7(6):1429–1439. 10.1038/mi.2014.32 PMC4205272 24781051

[4] Iyer S, Viernes DR, Chisholm JD, Margulies BS, Kerr WG (2014) SHIP1 regulates MSC numbers and their osteolineage commitment by limiting induction of the PI3K/Akt/beta-Catenin/Id2 Axis. Stem Cells Dev. 10.1089/scd.2014.0122 (in Eng) PMC4172390 24857423

[5] Iyer S, Margulies BS, Kerr WG (2013) Role of SHIP1 in bone biology. Ann N Y Acad Sci 1280:11–4. 10.1111/nyas.12091 PMC3670113 23551095

[6] Iyer S, Brooks R, Gumbleton M, Kerr WG (2014) SHIP1-expressing mesenchymal stem cell regulate hematopoietic stem cell homeostasis and lineage choice during aging. Stem cells and development. 10.1089/scd.2014.0501. Accessed 19 December 2014 PMC4403265 25525673

[7] Kerr WG, Park MY, Maubert M, Engelman RW (2011) SHIP deficiency causes Crohn’s disease-like ileitis. Gut 60(2):177–188. 10.1136/gut.2009.202283 PMC3022365 20940287

[8] Helgason CD et al (1998) Targeted disruption of SHIP leads to hemopoietic perturbations, lung pathology, and a shortened life span. Genes Dev 12(11):1610–1620 10.1101/gad.12.11.1610 PMC316868 9620849

[9] Paraiso KH, Ghansah T, Costello A, Engelman RW, Kerr WG (2007) Induced SHIP deficiency expands myeloid regulatory cells and abrogates graft-versus-host disease. J Immunol 178(5):2893–2900. 10.4049/jimmunol.178.5.2893 17312133

[10] Liu Q et al (1999) SHIP is a negative regulator of growth factor receptor-mediated PKB/Akt activation and myeloid cell survival. Genes Dev 13(7):786–791. 10.1101/gad.13.7.786 PMC316591 10197978

[11] Burnet M (1957) Cancer; a biological approach. I. The processes of control. Br Med J 1(5022):779–786. 10.1136/bmj.1.5022.779 PMC1973174 13404306

[12] Dunn GP, Old LJ, Schreiber RD (2004) The immunobiology of cancer immunosurveillance and immunoediting. Immunity 21(2):137–148. 10.1016/j.immuni.2004.07.017 15308095

[13] Schumacher TN, Scheper W, Kvistborg P (2019) Cancer neoantigens. Annu Rev Immunol 37:173–200. 10.1146/annurev-immunol-042617-053402 30550719

[14] Shankaran V et al (2001) IFNgamma and lymphocytes prevent primary tumour development and shape tumour immunogenicity. Nature 410(6832):1107–1111. 10.1038/35074122 11323675

[15] Smyth MJ, Thia KY, Street SE, MacGregor D, Godfrey DI, Trapani JA (2000) Perforin-mediated cytotoxicity is critical for surveillance of spontaneous lymphoma. J Exp Med 192(5):755–760. 10.1084/jem.192.5.755 PMC2193269 10974040

[16] Swann JB, Smyth MJ (2007) Immune surveillance of tumors. J Clin Investig 117(5):1137–1146. 10.1172/JCI31405 PMC1857231 17476343

[17] Davidson WF, Giese T, Fredrickson TN (1998) Spontaneous development of plasmacytoid tumors in mice with defective Fas-Fas ligand interactions. J Exp Med 187(11):1825–1838. 10.1084/jem.187.11.1825 PMC2212316 9607923

[18] Zerafa N et al (2005) Cutting edge: TRAIL deficiency accelerates hematological malignancies. J Immunol 175(9):5586–5590. 10.4049/jimmunol.175.9.5586 16237043

[19] Takahashi H et al (2006) FAS death domain deletions and cellular FADD-like interleukin 1beta converting enzyme inhibitory protein (long) overexpression: alternative mechanisms for deregulating the extrinsic apoptotic pathway in diffuse large B-cell lymphoma subtypes. Clin Cancer Res 12(11 Pt 1):3265–3271. 10.1158/1078-0432.CCR-06-0076 16740746

[20] Wohlfart S et al (2004) FAS (CD95) mutations are rare in gastric MALT lymphoma but occur more frequently in primary gastric diffuse large B-cell lymphoma. Am J Pathol 164(3):1081–1089. 10.1016/S0002-9440(10)63195-1 PMC1614721 14982861

[21] Smyth MJ, Dunn GP, Schreiber RD (2006) Cancer immunosurveillance and immunoediting: the roles of immunity in suppressing tumor development and shaping tumor immunogenicity. Adv Immunol 90:1–50. 10.1016/S0065-2776(06)90001-7 16730260

[22] Zorn E, Hercend T (1999) A MAGE-6-encoded peptide is recognized by expanded lymphocytes infiltrating a spontaneously regressing human primary melanoma lesion. Eur J Immunol 29(2):602–607. 10.1002/(SICI)1521-4141(199902)29:02<602::AID-IMMU602>3.0.CO;2-Y 10064076

[23] Zorn E, Hercend T (1999) A natural cytotoxic T cell response in a spontaneously regressing human melanoma targets a neoantigen resulting from a somatic point mutation. Eur J Immunol 29(2):592–601. 10.1002/(SICI)1521-4141(199902)29:02<592::AID-IMMU592>3.0.CO;2-2 10064075

[24] Ferradini L et al (1993) Analysis of T cell receptor variability in tumor-infiltrating lymphocytes from a human regressive melanoma. Evidence for in situ T cell clonal expansion. J Clin Invest 91(3):1183–1190. 10.1172/JCI116278 PMC288075 8450047

[25] Rooney MS, Shukla SA, Wu CJ, Getz G, Hacohen N (2015) Molecular and genetic properties of tumors associated with local immune cytolytic activity. Cell 160(1–2):48–61. 10.1016/j.cell.2014.12.033 PMC4856474 25594174

[26] Hodi FS et al (2010) Improved survival with ipilimumab in patients with metastatic melanoma. N Engl J Med 363(8):711–723. 10.1056/NEJMoa1003466 PMC3549297 20525992

[27] Pedersen N, Duricova D, Elkjaer M, Gamborg M, Munkholm P, Jess T (2010) Risk of extra-intestinal cancer in inflammatory bowel disease: meta-analysis of population-based cohort studies. Am J Gastroenterol Meta-Anal Rev 105(7):1480–1487. 10.1038/ajg.2009.760. (**(in Eng)**) 20332773

[28] Jess T, Gamborg M, Matzen P, Munkholm P, Sorensen TI (2005) Increased risk of intestinal cancer in Crohn’s disease: a meta-analysis of population-based cohort studies. Am J Gastroenterol Comp Study Meta-Anal Rev 100(12):2724–2729. 10.1111/j.1572-0241.2005.00287.x. (**(in Eng)**) 16393226

[29] Rubin DC, Shaker A, Levin MS (2012) Chronic intestinal inflammation: inflammatory bowel disease and colitis-associated colon cancer. Front Immunol 3:107. 10.3389/fimmu.2012.00107 PMC3347037 22586430

[30] Pohl C, Hombach A, Kruis W (2000) Chronic inflammatory bowel disease and cancer. Hepato-gastroenterol Rev 47(31):57–70. [Online].PMID: 10690586 http://www.ncbi.nlm.nih.gov/pubmed/10690586 10690586

[31] Sun X et al (2022) Inflammatory cell-derived CXCL3 promotes pancreatic cancer metastasis through a novel myofibroblast-hijacked cancer escape mechanism. Gut 71(1):129–147. 10.1136/gutjnl-2020-322744 33568427

[32] Trehan R et al (2025) SPP1 + macrophages cause exhaustion of tumor-specific T cells in liver metastases. Nat Commun 16(1):4242. 10.1038/s41467-025-59529-0 PMC12059142 40335453

[33] Bill R et al (2023) CXCL9:SPP1 macrophage polarity identifies a network of cellular programs that control human cancers. Science 381(6657):515–524. 10.1126/science.ade2292 PMC10755760 37535729

[34] Sultan H et al (2024) Neoantigen-specific cytotoxic Tr1 CD4 T cells suppress cancer immunotherapy. Nature 632(8023):182–191. 10.1038/s41586-024-07752-y PMC11291290 39048822

[35] Kelsall B (2008) Recent progress in understanding the phenotype and function of intestinal dendritic cells and macrophages. Mucosal Immunol 1(6):460–469. 10.1038/mi.2008.61 PMC4780321 19079213

[36] Efremova M et al (2018) Targeting immune checkpoints potentiates immunoediting and changes the dynamics of tumor evolution. Nat Commun 9(1):32. 10.1038/s41467-017-02424-0 PMC5750210 29296022

[37] Collazo MM et al (2009) SHIP limits immunoregulatory capacity in the T-cell compartment. Blood 113(13):2934–2944. 10.1182/blood-2008-09-181164 PMC2662640 19136659

[38] Collazo MM, Paraiso KH, Park MY, Hazen AL, Kerr WG (2012) Lineage extrinsic and intrinsic control of immunoregulatory cell numbers by SHIP. Eur J Immunol 42(7):1785–1795. 10.1002/eji.201142092 PMC3816569 22535653

[39] Somasundaram R et al (2017) Analysis of SHIP1 expression and activity in Crohn’s disease patients. PLoS One 12(8):e0182308. 10.1371/journal.pone.0182308 PMC5540589 28767696

[40] Fernandes S et al (2018) SHIP1 deficiency in inflammatory bowel disease is associated with severe Crohn’s disease and peripheral T cell reduction. Front Immunol 9:1100. 10.3389/fimmu.2018.01100 PMC5972310 29872435

[41] Fuhler GM et al (2012) Therapeutic potential of SH2 domain-containing inositol-5’-phosphatase 1 (SHIP1) and SHIP2 inhibition in cancer. Mol Med 18(1):65–75. 10.2119/molmed.2011.00178 PMC3269644 22033675

[42] Ong CJ et al (2007) Small-molecule agonists of SHIP1 inhibit the phosphoinositide 3-kinase pathway in hematopoietic cells. Blood 110(6):1942–1949. 10.1182/blood-2007-03-079699 17502453

[43] Pedicone C et al (2022) Discovery of a novel SHIP1 agonist that promotes degradation of lipid-laden phagocytic cargo by microglia. iScience 25(4):104170. 10.1016/j.isci.2022.104170 PMC9020084 35465359

