# Supplementary material for: SHIP1 is required for T cell surveillance of occult malignancies
Source: Cell Mol Life Sci. 2026 Jul 7;83(1):352. doi: 10.1007/s00018-026-06326-7 (PMC13627489; doi:10.1007/s00018-026-06326-7)
Supplement: Supplementary file 1 [file 18_2026_6326_MOESM2_ESM.pdf]

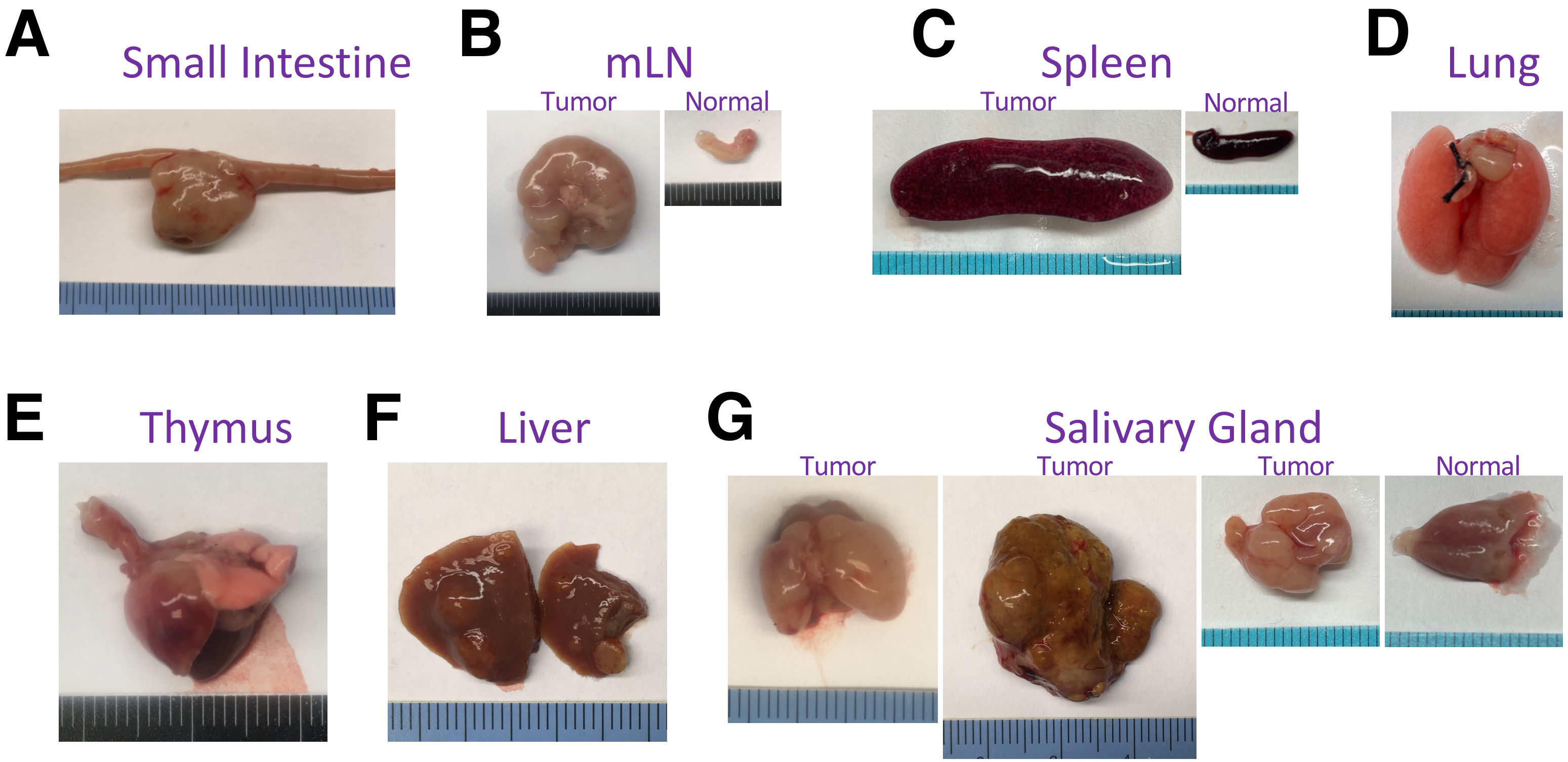


*Fig.S1 Fernandes et al*

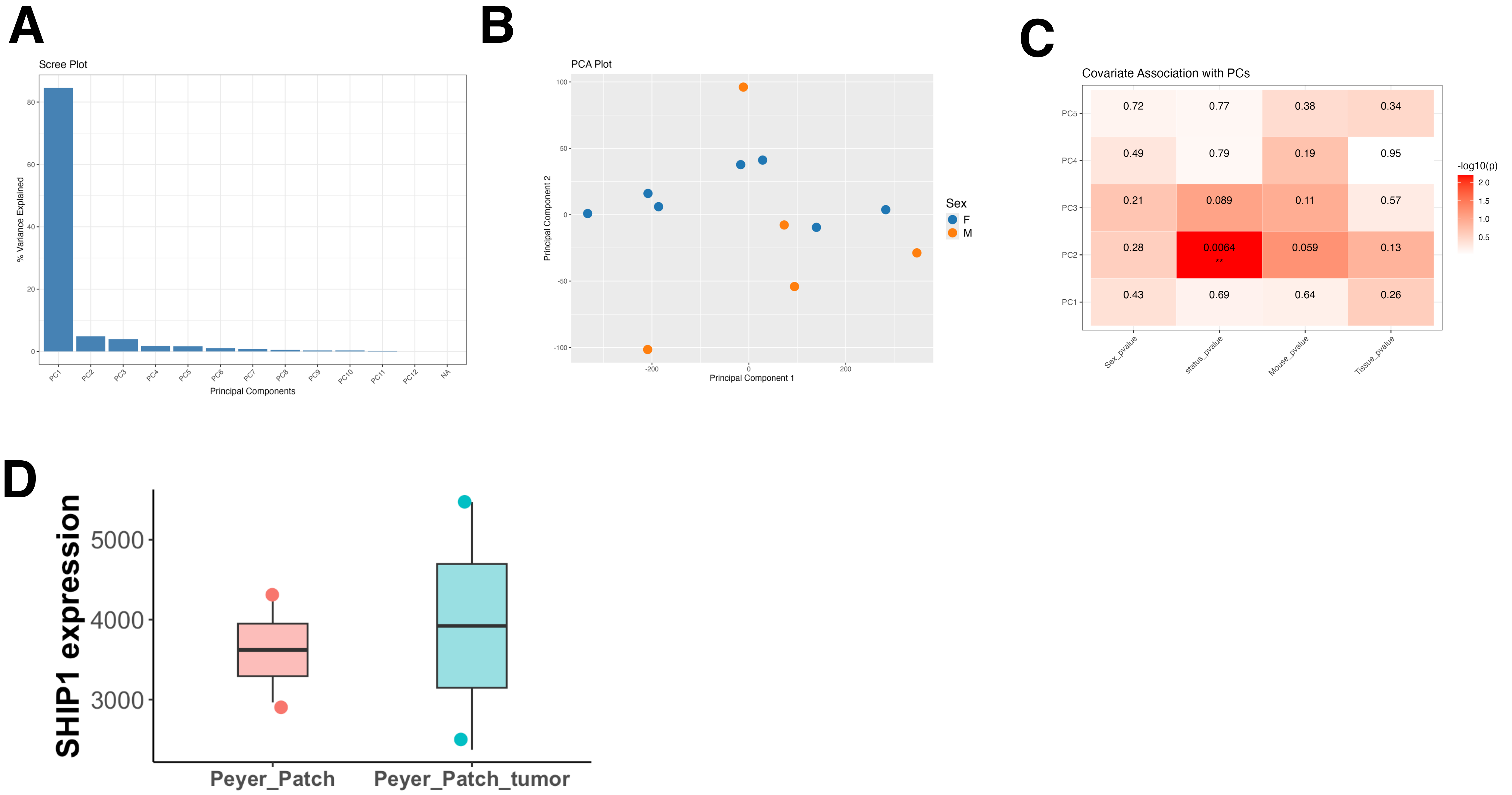


*Fig. S2 Fernandes et al*

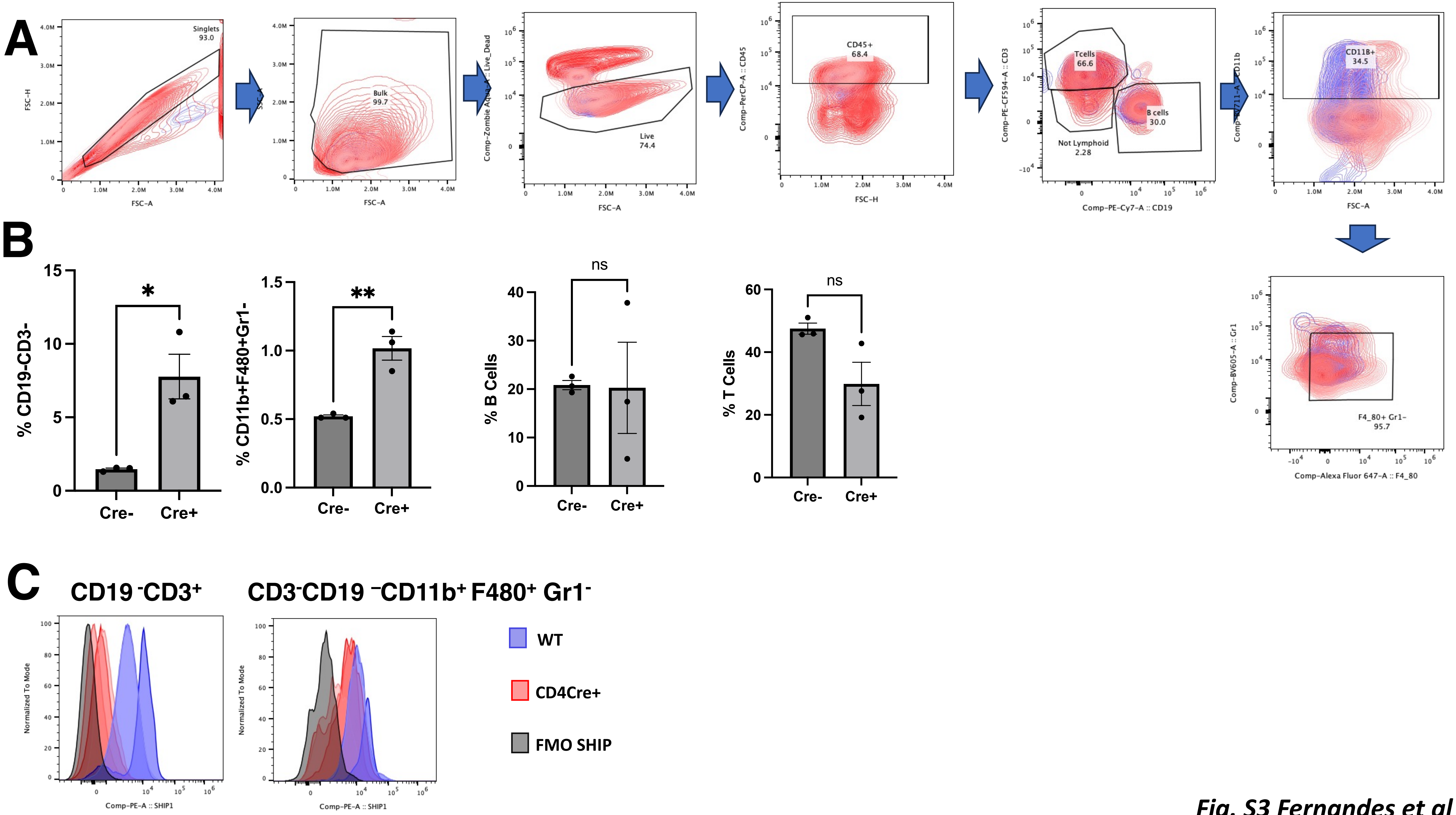


*Fig. S3 Fernandes et al*
